# Judicial consequences in Spain for the completion of the medical death certificate

**DOI:** 10.1007/s00414-021-02733-6

**Published:** 2021-10-26

**Authors:** Pilar Pinto Pastor, Enrique Dorado Fernández, Benjamín Herreros, Elena Albarrán Juan, Andrés Santiago-Sáez

**Affiliations:** 1 Forensic Medicine, Medico-Legal Institute of Madrid, Madrid, Spain; 2 Departamento de Medicina Legal, Psiquiatría y Patología. Facultad de Medicina, Universidad Complutense de Madrid, Pza. Ramón Y Cajal, s/n. 28040, Madrid, Spain; 3 Hospital Universitario Fundación (Internal Medicine Unit), Alcorcón, Madrid Spain; 4 Primary Care Medicine, Móstoles, Madrid Spain; 5 Clínico San Carlos Hospital (In-Hospital Legal Medicine Unit), Madrid, Madrid Spain

**Keywords:** Medical certificate of cause of death, Medico-legal documents, Jurisprudence

## Abstract

The completion of the death certificate is indispensable in Spain for a death to be recorded in the civil registry. Occasionally, doctors may be reluctant to sign a death certificate due to possible legal consequences. This study seeks to analyse the possible judicial consequences doctors may face upon filling out this medico-legal document. Sentences published on the Judicial Power’s website between 2009 and 2019 containing the term “death certificate” were analysed. From a total of 2100 sentences examined, only 15 were found to contain the term “death certificate” as part of the claim. In only 7 of these cases the claim was made against the physician, and in 5 the physician was found guilty. Three of them concluded falsity via criminal proceedings, one via administrative proceedings for refusing to sign the certificate and one through civil proceedings for filling out an erroneous antecedent cause of death. In view of the above, it can be inferred that the completion of the death certificate poses few judicial consequences for physicians. In addition, this study reveals the importance of the death certificate document as evidence in judicial proceedings.

## Introduction

The death certificate (DC) is an official document whose main function is to serve as a reference for public health policies regarding the causes of death. In addition, it is a necessary document to register the death of an individual and authorize the burial [1–11].

In Spain, the DC is used in the Civil Registry to record a person’s decease [12, 13] and, on the other hand, is used by the national statistics institute (Instituto Nacional de Estadística—INE) to reregister the national statistics of the cause of death [14–16]. In Spain, the DC must be used in all deaths in which there is no suspicion of violence or criminality involved [12]. Actually, any case without DC becomes a judicial case and requires a legal investigation. The completion of the DC is part of the doctor’s healthcare activities integrated in the end-of-life care [5, 17]. Beyond the ethical responsibility, a doctor has also a deontological and legal obligation to complete it.

Various studies worldwide [1–4, 7, 10, 18–20] have proven it is common that DC have poor quality and present mistakes that hinder their interpretation for public health statistical purposes, such as the use of physiologic processes (i.e. cardiorespiratory arrest) instead of the causes, mistakes in the sequence of causes or deficits in recording the evolution of the process. For this reason, many reviews have stated the importance of a correct exploration of the corpse, using the official WHO forms, training of professionals involved in filling out the certificate [6, 18]and the use of electronic format if possible.

Due to one cause or another, the fact is that the signing of the DC is a procedure that causes reluctance and insecurity in many professionals [7]. In Spain, the situation is not different due to the possible judicial consequences in the case of, for instance, a mistake in determining the cause of death [13]. The Spanish DC document is filled in by physicians when there is no suspicion of a violent manner of death. In natural deaths, it should be completed if the medical records of the patient allow to infer the cause of death. On the contrary, in cases of sudden death or any suspicion about the death circumstances the DC should not be filled out. Any death case lacking the DC becomes a legal case and, consequently, a judicial investigation is opened. Physicians’ reticence for signing the DC turns natural deaths into legal cases. Despite the fact that since 2011 the doctor's obligation to complete this medico-legal document is stated in article 36.6 of the Code of Ethics [21], conflicts and reluctance still exist, especially in the out-of-hospital context.

Several authors state that it is not likely for a doctor who, having examined a corpse, fills out the DC in good faith, to be taken to court; even in the case that he/she is mistaken in the cause of death or if, finally, it turns out not to be due to natural causes [13, 22, 23]. Nevertheless, there is a lack of studies, at least in our field, that investigate the judicial consequences for doctors regarding the DC. The relevance of this study lies in the fact that it carries out a comprehensive analysis of the existing court sentences gathered in CENDOJ^1^ between 2009 and 2019 (inclusive) containing the term “death certificate”. This analysis determines the number of cases in which doctors are prosecuted in relation to the content of the medical DC, how many of them are sentenced, the judicial sphere and the reason for the sentence. The purpose is to determine whether judicial consequences exist for doctors filling out the DC in case of an error in the cause of death or any other related reason.

The goal of this research is to provide legal certainty and support for doctors completing the medical DC, which after all is a medical action that should not be avoided [14]. We must bear in mind that the consequences of not completing the medical DC include the judicialization of the corpse, putting the family through a process with emotional, legal and even economic repercussions [12, 24].

## Material and methods

A retrospective longitudinal study has been carried out in which the official sentence searcher of the Judicial Power site was revised for all sentences containing the term “death certificate” in the period between January 1^st^ 2009 and December 31^st^ 2019. This term was chosen instead of “medical death certificate”, because a preliminary evaluation led to the conclusion that jurists referred to the document as “Death Certificate” and not as “Medical Death Certificate”.

The main hypothesis of the research is that the mistakes committed by doctors in the completion of the causes of death in the DC pose no consequences for them. Likewise, the cases in which doctors were prosecuted and the DC was involved in the claim were examined in order to determine if they resulted in condemnatory sentences or acquittals, under which jurisdiction they occurred and, in the case of a conviction, what was its motive.

A shortcoming of the study resides in the fact that the CENDOJ is obliged to collect the sentences by the Provincial High Court, the High Court of Justice, the National High Court, the Supreme Court and the Constitutional Court. But the sentences by lower instances (such as the Magistrate’s Court, or Trial Court, the Criminal Court, the Court of First Instance, the Labour Court) are not registered unless the judge responsible for the case voluntarily introduces the sentence in the browser. Therefore, it was not possible to include in the study all the processes that did not reach the higher instances. Another of the study’s limitations is that when the DC is mentioned in the sentences, the term is used indiscriminately to refer to three different documents/situations: the medical death certificate which is the real object of this study, the forensic autopsy reports in the case of judicialized cases and the literal death certificate^2^ issued by the Civil Registry for a legal procedure at the request of one of the parties concerned. The judicial cases have been excluded from the analysis. Regarding the sentences which make reference to the literal death certificate, they have been included in the research carried out, because the content reflected is obtained from the medical DC and not including it would imply downplaying the importance of the medical DC and its content for the subsequent procedures carried out with the information they contain.

In addition, with regard to contextualising the results, the number of annual deaths has been searched in the INE (National Statistics Institute) database.

The data collection sheet included the following variables included in Table 1.

**Table 1:** Resume of the data collection sheet

Identifying data of the STC	DC data	Cases that specifically claim the content of the DC or problems with its issue
Year	Only the DC is mentioned	Jurisdictional scope
Autonomous Community (Regional State)	The content of the DC is mentioned as a necessary part in the STC	Subject of the claim
Jurisdictional scope	DC that addresses medical malpractice	Reason for the claim
	Cases that specifically claim the content of the DC or problems with its issue	Conviction or acquittal

For the statistical analysis, a descriptive study of frequencies and percentages has been carried out for each of the variables studied.

## Results

A total 2100 STC were studied within a period of 11 years, from 2009 to 2019 inclusive. The number of sentences mentioning the death certificate showed an increasing tendency within the period in study (Table 2).

**Table 2:** Cases analysed per year in relation to the total (*n* = 2100)

Year	Total deaths	Number of STC	Rate of DC included in STC * 100,000 deaths	Percentage of total researched sentences
2009	384.933	159	41.30	7.57%
2010	382.047	181	47.37	8.61%
2011	387.911	180	46,40	8.57%
2012	402.950	178	44.17	8.47%
2013	390.419	190	48.66	9.04%
2014	395.830	198	50.02	9.42%
2015	422.568	167	39.52	7.95%
2016	410.611	201	48.95	9.57%
2017	424.523	220	51.82	10.47%
2018	424.523	220	51.82	10.47%
2019	418.703	206	-	9.8%

**Fig. 1:**
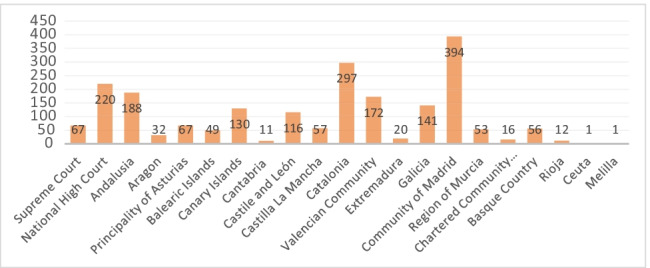
Diagram representing the number of STC analysed per Autonomous Community

Figure 1 shows number of STC analysed at a national level (Supreme Court and National High Court) and in each Autonomous Community (Regional States).

**Fig. 2:**
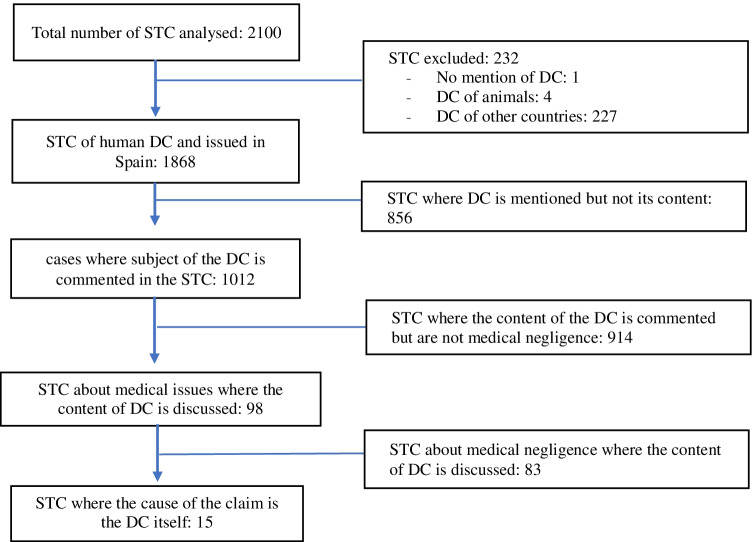
Diagram of the number of sentences (STC) analysed containing the term death certificate (DC)

Aimed at studying those cases in which the motive of the judicial claim was solely or partially based on the content of the death certificate, the STC which did not have a motive related to such document were gradually discarded (Fig. 2).

From all the STC analysed, 232 were excluded: for not including the content about the DC (1 case), for not being about human deaths (4 cases of animal DC) or for being DC completed abroad (227 STC). Among the cases not excluded, there were 24 STC that made reference to judicial cases in which the DC was therefore not issued, but an advance report on the autopsy instead.

**Fig. 3:**
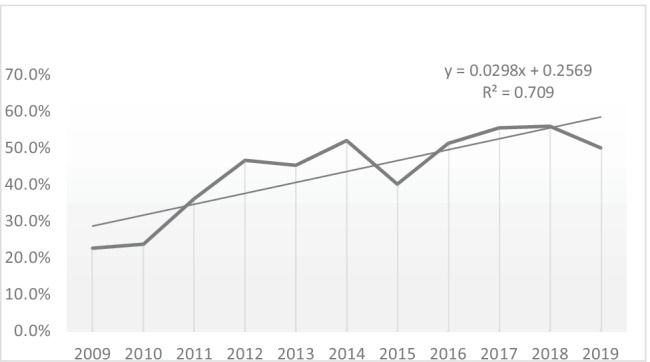
Representation of % of STC commenting the DC/total yearly STC

In 856 of the STC analysed, the DC is mentioned but there is no reference made to its content. Within this group, 8 cases were related to medical negligence. Part of the content of the DC is mentioned in 914 occasions, not being cases of medical malpractice. There is an increasing tendency in the total yearly STC of comments on the content of the DC in those STC analysed (Fig. 3), though it is more pronounced in cases not related with medical professional responsibility than in cases that do have a motive related to it (Table 3).

**Table 3:** STC naming or commenting part of the DC content

Year	STC commented/total STC in year (malpractice cases)	STC commented/total STC in year (not malpractice)	% of STC commented with regard to total STC per year
2009	3/159	34/159	22.64%
2010	9/181	37/181	23.75%
2011	6/180	63/180	36.11%
2012	7/178	77/178	46.62%
2013	13/190	82/190	45.26%
2014	10/198	99/198	52.02%
2015	10/167	67/167	40.11%
2016	10/201	103/201	51.24%
2017	6/220	123/220	55.45%
2018	2/220	124/220	55.90%
2019	7/206	105/206	50%
Total	83/2100	914/2100	47.47%

Finally, in 15 of the analysed STC, the motive for the claim is, fully or partially, the DC, as can be appreciated in Table 4.

**Table 4:** Summary of STC in which the DC was judged

Year	Autonomous Community (Regional State)	Jurisdictional organ	Scope	Claim against	Meaning of the STC	Object being judged
2009	-	National High Court	Criminal law	Military doctors	Sentenced	DC and necropsy reports forgery (Yak-42)
2010	-	Supreme Court	Criminal law	Military doctors	Sentenced	The National High Court STC is confirmed (appeal of the Yak-42 STC)
2012	Valencian Community	Provincial High Court	Civil law	Emergency doctor	Acquitted	Alteration of the cause of death in the DC in a case of malpractice
2013	Principality of Asturias	High Court of Justice	Contentious-administrative jurisdiction	Primary doctor	Acquittal	Refusal for 5 h to sign the MDC. Not found guilty because the delay was due to the institutional entity and not the doctor's responsibility
2013	Castile and Leon	High Court of Justice	Contentious-administrative jurisdiction	Emergency doctor	Sentenced	Refused to sign the DC even though the medical history explained the death
2016	Catalonia	High Court of Justice	Social law	Social Security Treasury	Acquittal	Wrong name of the deceased in the DC. This is not the main motive for the claim, it is included in the case. The change of name is agreed
2017	Galicia	Regional Military Court	Military law	Sergeant – relative of the deceased	Sentenced	Forgery of the year of the DC to delay the admission into the academy
2018	Galicia	Provincial High Court	Criminal law	Daughter of the deceased	Sentenced	Forgery of the year of death to collect the retirement pension (between 2002 and 2015)
2018	Galicia	Provincial High Court	Civil law	Relative of the deceased	Sentenced	Request to a doctor to forge the time of the DC in order to withdraw 18,000€ from the deceased's account. The physician changes the DC without checking necessary procedures. Physician is cleared
2018	Santa Cruz de Tenerife	Provincial High Court	Criminal law	Mortuary worker	Sentenced	Forgery of hour of the DC in order to carry out the burial within 24 h of death
2018	Community of Madrid	High Court of Justice	Social law	Social Security Treasury	Sentenced	Asbestosis is claimed as cause of death as well as the fact it was not registered in the DC. The Court rejects the second part of the claim
2019	Community of Madrid	Provincial High Court	Criminal law	Swindlers	Sentenced	Several swindlers forge DC, among other documents, to convince their victims of a false inheritance and request money for necessary previous legal procedures
2019	Catalonia	Provincial High Court	Civil law	Nursing home doctor and nursing home	Sentenced	Reports fictitious contagious disease on DC. Request for moral damage
2019	Community of Madrid	Provincial High Court	Criminal law	Neurosurgeon	Sentenced	Intentionally alters cause of death to avoid possible professional responsibility due to malpractice
2019	Castile and Leon	High Court of Justice	Contentious-administrative jurisdiction	College of Physicians	Sentenced	Charged an extra cost for issuing the medical DC for three years running, which solely benefitted the College

One of the unexpected findings of the study was how the DC fulfils an important role as a means of proof in the analysed STC. Of the 2100 STC analysed, in 47.47% part of the DC is commented on in relative depth. If the 15 cases in which the DC is the main motive for the claim are included, the percentage rises to 48.19%. We have also analysed which part of the document is most commented upon in the sentences, finding that the most commonly gathered data in the STC is about the death.

Table 5 collects the main aspects commented on in these sentences. It also includes information about how many of them were not DC but judicialized deaths as well as the cases in which it was possible to determine it was referring to the *literal death certificate* issued by the Civil Registry.

**Table 5:** Features commented in the STC

Data commented in the STC	Number
Identity	4
Data about the death	383
Place of death	134
Cause of death and medical history	139
Civil status	56
Nationality	5
Daughter, son of…	3
Age of deceased	5
More than one datum	147
Judicialized deaths	24
Literal death certificate (Civil Registry)	28

## Discussion

The DC has been proven to be a document that generates conflicts for doctors around the world. However, its importance as a necessary document for health policies requires the implication of professionals. In Spain, practicing physicians often show reluctance to sign the DC, a situation that requires a judicialization of natural deaths. This not only entails a waste of public resources but also emotional, legal and even economic consequences for the family. In those natural deaths with a medical history in which the physician does not assume the responsibility of signing the DC, it can be considered that the professional is acting against general ethics, ethics of the medical profession and legality. The cause of death in the DC is a presumptive diagnosis and its purpose is statistical. However, one of the arguments used most commonly by physicians to refuse to sign the DC is the lack of certainty about the cause of death and the possible legal consequences that can arise from a mistake in registering said cause of death. This study intends to shed light on the possible legal consequences related to filling out the DC. With this purpose, it examines all the sentences gathered in the CENDOJ directory containing the term “death certificate” within the period between 2009 and 2019 (inclusive). As a limit of this study, we found that it is only mandatory to include in the CENDOJ registry the sentences of High Courts, while Low court sentences are only included if the judge chooses to do so. Thus, though most of the cases are appealed and therefore reach a high instance and are consequently included in the CENDOJ; nevertheless, some cases may be lost in this research.

Regarding the 15 sentences found in which the DC was being tried as either the main part or an accessory of the proceedings, it is true that only in 7 of those sentences the party under claim was the physician. In this group, the case of the Yakolev 42 [25] air accident appears twice: in the criminal proceedings at the National High Court and later in the appeal of the same process at the Supreme Court.^3^ We are therefore referring to 6 cases (although there are 7 sentences) in which 4 were sentenced as guilty and 2 were acquitted. The motives for the sentences were intentional forgery of the document in the Yak-42 case and forgery of a cause of death by a neurosurgeon to avoid a case of possible medical negligence. Another case was sentenced as guilty because a physician refused to sign the DC and did not even check the medical history of the deceased to learn if there existed a cause that explained a natural death. In this case, the doctor alleged that the cause of death was unknown, but he did not request a clinical autopsy to be conducted. That is to say, it was an unfounded refusal to perform a medical action to which patients and relatives have a right. In this case, an emergency service colleague ultimately signed the document but the emergency room physician who refused to sign the DC was sentenced for serious misconduct. Finally, one physician (social-sanitary centre) was sentenced for registering an infectious-contagious history in the DC when actually the patient did not have any infectious illness. In this case, the sentence established a fine of 1000€. This case shows the importance of studying the patient’s medical history and ensuring it is up to date as a previous step to signing the DC.

Other information relevant to the study can be extracted from these STC. On one hand, the time of a DC must not be modified or forged in order to bring forward the burial or for any other reason. In fact, the DC is a medico-legal document, and its intentional modification can involve criminal liability. On the other, it is frequent to find references about the double function of the DC as a medico-legal document necessary to register the death of a person in the Civil Registry as well as a statistic to record the cause of death for public health data.

However, our study shows another function of the DC not very appreciated until now: its importance as a means of proof in judicial proceedings. The DC is used in 47% of the STC to certify any of the items that appear in the document. Most frequently, the elements gathered are the data about the death, the cause of death, the medical history and the place of death. In 147 cases, more than one item from the DC was mentioned.

In all the cases in which the content of the DC was commented in the STC, the ones in which the judicial claim was about possible medical negligence are especially relevant at a healthcare level. Of the total 91 STC related to professional malpractice, 83 of them collected or commented part of the DC content in the STC itself.

It can be concluded that mistakes in filling out the DC do not carry legal consequences for the doctors who sign them as long as the document is not altered intentionally. The collection of the causes of death after the examination of the corpse and the review of the medical history will be carried out as a presumptive diagnosis. Therefore, a mistake in the procedure will not entail legal consequences for the doctor based on the analysis performed.

It is essential to provide further training to doctors about the DC to ensure they understand it is a necessary requirement which must be filled out satisfactorily and to reduce their reluctance and the possible medico-legal consequences entailed [26].

The imminent implementation of the digital DC in Spain [12, 27] will pose a new challenge for professionals as it will require specific training. However, as long as the truthfulness of the document is preserved and professional responsibilities are not eluded, it can be concluded that the completion of the DC is neither a source of professional responsibility nor a cause for condemnatory sentences for physicians.

Lastly, we consider it advisable that jurists adapt the terminology specifying the situations in which they refer to the medical death certificate or to the literal death certificate, instead of indiscriminately using the term death certificate.

## Data Availability

All the material reviewed during the study is available at www.cendoj.es.

## Footnotes

1 The Judicial Documentation Centre (CENDOJ) is the technical body of the General Judicial Power Council (CGPJ) in charge of the official issue of jurisprudence. Through this website, the sentences and resolutions by the Supreme Court, the National High Court, High Courts of Justice and Provincial High Courts are provided freely to the citizenry (https://www.poderjudicial.es/search/indexAN.jsp).

2 Literal death certificate is a document issued by the Civil Registry once the DC has already been recorded and includes the basic information of the decease (name, date and place of death, age, ID number…) but, in Spain, it does not include the cause of death because this data is only used for public health purpose.

3 The Yak-42 case was a military plane accident that occurred on May 26^th^, 2003. Seventy-five people were killed. The autopsy and identification in this case was carried out in equal parts by a Spanish military team and a Turkish one. In fact, the Yakolev case was a violent death but in the sentence the forgery of the medico-legal documents were all the time referred to as a forgery of the DC.
